# Evaluating the performance characteristics of different antimicrobial susceptibility testing methodologies for testing susceptibility of gram-negative bacteria to tigecycline

**DOI:** 10.1186/s12879-021-06338-7

**Published:** 2021-07-27

**Authors:** Sima Babaei, Mehri Haeili

**Affiliations:** Department of Animal Biology, Faculty of Natural Sciences, University of Tabriz, Tabriz, Iran

**Keywords:** Tigecycline, Broth microdilution, Etest, Agar dilution, Disk diffusion, Gram-negative bacteria

## Abstract

**Background:**

The current emergence of multi-drug resistance among nosocomial pathogens has led to increased use of last-resort agents including Tigecycline (TGC). Availability of reliable methods for testing TGC susceptibility is crucial to accurately predict clinical outcomes. We evaluated the influence of different methodologies and type of media on TGC susceptibility of different gram-negative bacteria of clinical origin.

**Methods:**

The TGC susceptibility of 84 clinical isolates of *Klebsiella pneumoniae* (*n* = 29), *Escherichia coli* (*n* = 30), and *Acinetobacter baumannii* (*n* = 25) was tested by broth microdilution (BMD), Etest, agar dilution (AD) and disk diffusion (DD) methods using Mueller Hinton agar from Difco and Mueller Hinton broth (MHB) from two different manufacturers (Difco and Condalab). FDA TGC susceptibility breakpoints issued for Enterobacteriaceae were used for interpretation of the results.

**Results:**

MICs determined by BMD using MHB from two suppliers showed a good correlation with overall essential agreement (EA) and categorical agreement (CA) being 100% and 95% respectively. However, a twofold rise in BMD-Condalab MICs which was detected in 50% of the isolates, resulted in changes in susceptibility categories of few isolates with MICs close to susceptibility breakpoints leading to an overall minor error (MI) rate of 4.7%. Among the tested methods, Etest showed the best correlation with BMD, being characterized with the lowest error rates (only 1% MI) and highest overall EA (100%) and CA (98.8%) for all subsets of isolates. AD yielded the lowest overall agreement (EA 77%, CA 81%) with BMD in a species dependent manner, with the highest apparent discordance being found among the *A. baumannii* isolates. While the performance of DD for determination of TGC susceptibility among Enterobacteriaceae was excellent, (CA:100% with no errors), the CA was lower (84%) when it was used for *A. baumannii* where an unacceptably high minor-error rate was noted (16%). No major error or very major error was detected for any of the tested methods.

**Conclusions:**

Etest can be reliably used for TGC susceptibility testing in the three groups of studied bacteria. For the isolates with close-to-breakpoint MICs, testing susceptibility using the reference method is recommended.

## Introduction

Tigecycline (TGC) is a new semisynthetic glycylcyclin with expanded-spectrum antibacterial activity against gram-negative and gram-positive bacteria [1]. It retains activity against most clinically significant multi-drug resistant (MDR) gram-negative bacteria (GNB) including extensively drug resistant *A. baumannii* [2] and carbapenem resistant Enterobacteriaceae (CRE) [3, 4]. Tigecycline is approved by the FDA for complicated skin and skin structure infections, complicated intra-abdominal infections, and community-acquired bacterial pneumonia [5]. TGC inhibits bacterial protein synthesis and is not affected by the known tetracycline resistance determinants (ribosomal protection and TetA-E Efflux pumps) [6, 7]. Increased administration of TGC in clinical settings has resulted in the development of resistance to this last resort antibiotic among the difficult to treat pathogens. The mechanism of resistance to TGC primarily involves overexpression of RND family efflux pumps [8, 9], or it can be mediated by other determinants including *rpsJ* [5] and *tetA* mutations [10] or by enzymatic inactivation by TetX variants [11]. Despite being used clinically since 2005, TGC susceptibility testing is still considered challenging. There are currently no defined CLSI susceptibility breakpoints for tigecycline and EUCAST TGC breakpoints are only available for a handful of GNBs (*E. coli* and *C. koseri*). It has been demonstrated that the TGC susceptibility testing outcomes can be influenced by different factors including the chosen testing methodology and the manganese content or the age of the medium used for antimicrobial susceptibility testing (AST) [12–14]. The uptick in clinical use of tigecycline necessitates availability of reliable susceptibility testing methods as alternatives to the standard broth microdilution method. A simpler assay that could reliably test TGC susceptibility such as disk diffusion may be the only option in resource-limited clinical laboratories where performance of an ideal standardized microdilution assay is not practical. Similarly, Etest strips are a convenient alternative to conventional dilution-based susceptibility testing methods. The aim of this study was to assess the influence of different methodologies and type of medium on TGC susceptibility of different gram-negative bacteria of clinical origin.

## Materials and methods

### Bacterial isolates

The study included 84 clinical isolates of *Escherichia coli* (*n* = 30), *Klebsiella pneumoniae* (*n* = 29) and *A. baumannii* (*n* = 25) recovered during 2015 to 2020 from patients in four hospitals located in different regions of Iran. Identification of the isolates to species level was performed by conventional biochemical tests [15], detection of *bla*_OXA-51-like_ gene for *A. baumannii* [16] or Vitek2. Some of the tigecycline non-susceptible isolates were intentionally included from our previous works ( [17, 18] to have a complete series of susceptible (S), Intermediate (I) or resistant (R) isolates to assess the performance of different testing methods for correct identification of all three susceptibility categories. *E. coli* ATCC 25922 was used as quality control strain for antimicrobial susceptibility testing.

### Susceptibility testing

MICs were determined in triplicate by broth microdilution testing using freshly prepared (less than 12 h old) Mueller Hinton broth (MHB) from Difco (BD Diagnostic Systems, Sparks, MD) (lot 8,170,915) and Condalab (Madrid, Spain) (CAT:1214.00, Batch No. 802083). TGC concentrations (Glentham Life Sciences (UK) (batch No.176ZIJ and 389SOI) spanned a doubling dilution range of 0.015 mg/l to 64 mg/l. MICs were recorded after incubation at 35–37 °C in a non-CO_2_ incubator for 16 to 20 h as the lowest concentration of the agent that inhibited visible growth of the tested isolate as judged by the naked eye. Etest, disk diffusion and agar dilution were all performed using the Mueller-Hinton agar (MHA) from Difco (BD Diagnostic Systems, Sparks, MD) (lot 7,271,779) which have been previously determined to have a lower manganese content compared to other brands [13]. Agar diffusion methods were performed using Liofilchem Etest Strips (Liofilchem, Roseto degli Abruzzi, Italy) code 92144) containing concentration gradient range of TGC (0.016 to 256 mg/l) or disks containing 15 μg of TGC (Mast Co, Merseyside, UK) respectively according to the manufacturers’ instructions with incubation at 35 °C in the incubator for 16 to 24 h. The Etest MIC endpoint was read where the growth inhibition ellipse intersected the MIC scale on the Etest strip. Agar dilution was performed by addition of appropriate amounts of 25, 2.5 or 0.25 mg/ml stock solutions of TGC to molten sterile MHA to provide concentrations ranging from 0.03 to 64 mg/1 according to a method described previously [19]. MICs were recorded as the lowest concentration of the agent that inhibited visible growth of the tested isolate, disregarding the growth of a single colony or faint film caused by the inoculum.

### Interpretation of results and data analysis

Susceptibility results were compared and categorized in relation to BMD testing performed using MHB from Difco. Etest MIC values were rounded up to the next concentration of the standard doubling dilution scale when necessary. Categorical agreement (CA), essential agreement (EA), major errors (ME), very major errors (VME), and minor errors (MI) were evaluated as follow: EA was calculated by determining the number of test results that were within ±1 doubling dilution of the MIC obtained by BMD. CA was defined as the number of tests with correct susceptibility categorization between the method under evaluation and BMD. A very major error (VME) indicated a false-susceptible result; a major error (ME) indicated a false-resistant result; Minor errors (MI) were defined as one result yielding an intermediate category and the other either a susceptible or resistant result [20]. The statistical analyses were performed using MedCalc software, version 13 (MedCalc, Ostend, Belgium).

Due to the lack of established CLSI breakpoints for TGC at this time, Food and Drug Administration (FDA) breakpoints issued for *Enterobacteriaceae*, (susceptible ≤2 mg/l, intermediate = 4 mg/l, resistant ≥8 mg/l for MIC methods, and ≥ 19 mm, susceptible;15–18 mm, intermediate; ≤14 mm, resistant for disk diffusion) (https://www.accessdata.fda.gov/drugsatfda_docs/label/2013/021821s026s031lbl.pdf) were applied for interpretation of results. Since no TGC MIC breakpoints was available for *Acinetobacter* spp. we used the same breakpoints defined by the FDA for Enterobacteriaceae for interpretation of susceptibility testing results obtained for *A. baumannii*.

## Results

**Fig. 1:**
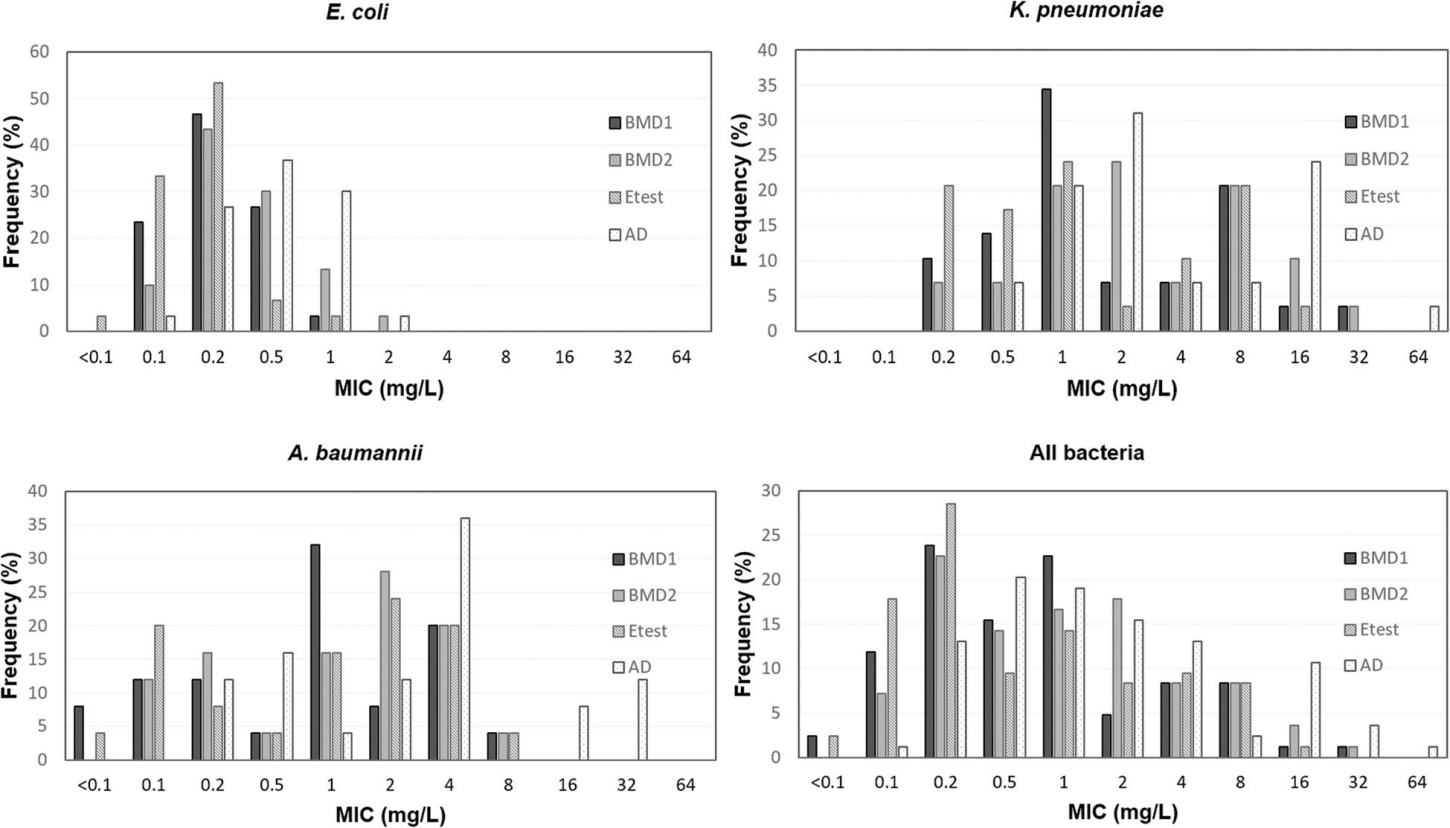
Distribution of MICs determined by four different dilution-based methods for all subsets of isolates (BMD1, Broth microdilution -Difco; BMD2, Broth microdilution -Condalab, AD, Agar dilution)

The TGC MIC measurements for the tested ATCC reference strain were within the acceptable quality control ranges as specified by the CLSI (BMD-Difco: 0.06 mg/l, BMD-Condalab: 0.12 mg/l, E-test: 0.06 mg/l, AD: 0.12 mg/l, DD: 25 mm). According to TGC susceptibility testing results obtained by BMD (Difco) as reference method, 68 isolates (30 *E. coli*, 19 *K. pneumoniae* and 19 *A. baumannii*) were TGC-susceptible and the remaining 16 isolates were found to be TGC non-susceptible (R (*n* = 9) or I (*n* = 7)) which included 10 *K. pneumoniae* (*n* = 8 resistant, *n* = 2 intermediate) and 6 *A. baumannii* (*n* = 1 resistant, *n* = 5 intermediate). Since some of the TGC-I or TGC-R isolates were included in the study on purpose, the obtained data does not reflect the true efficacy of TGC on the tested bacterial isolates. The TGC MIC distributions of the *E. coli*, *K. pneumoniae* and *A. baumannii* ranged from 0.1 to 1 mg/l, 0.2–32 mg/l and < 0.1–8 mg/l respectively. Table 1 and Fig. 1 show the TGC MIC distribution of the tested isolates determined using the different AST methods.

**Table 1:** Tigecycine MICs of isolates obtained by different testing methodologies or medium types

		Number of isolates with MICs (mg l^− 1^)										
Organism (n)	Method	<0. 1	0.1	0.2	0.5	1	2	4	8	16	32	64
All isolates (84)	BMD1	2	10	20	13	19	4	7	7	1	1	0
	BMD2	0	6	19	12	14	15	7	7	3	1	0
	Etest	2	15	24	8	12	7	8	7	1	0	0
	AD	0	1	11	17	16	13	11	2	9	3	1
*K. pneumoniae* (29)	BMD1	0	0	3	4	10	2	2	6	1	1	0
	BMD2	0	0	2	2	6	7	2	6	3	1	0
	Etest	0	0	6	5	7	1	3	6	1	0	0
	AD	0	0	0	2	6	9	2	2	7	0	1
*E. coli* (30)	BMD1	0	7	14	8	1	0	0	0	0	0	0
	BMD2	0	3	13	9	4	1	0	0	0	0	0
	Etest	1	10	16	2	1	0	0	0	0	0	0
	AD	0	1	8	11	9	1	0	0	0	0	0
*A. baumannii* (25)	BMD1	2	3	3	1	8	2	5	1	0	0	0
	BMD2	0	3	4	1	4	7	5	1	0	0	0
	Etest	1	5	2	1	4	6	5	1	0	0	0
	AD	0	0	3	4	1	3	9	0	2	3	0

*BMD1* Broth microdilution –Difco, *BMD2* Broth microdilution -Condalab, *AD* Agar dilution

### Comparison of BMD results performed by MHB from two different manufacturers

Overall, for half of the strains (50%), MICs determined by MHB from Condalab were 2-fold dilutions higher than those determined by BMD from Difco. Despite this, a good correlation was found for MIC values obtained by MHB from two suppliers with EA and CA being 100 and 95% respectively (Weighted kappa = 0.911, SE = 0.043, 95% CI = 0.826–0.995). There was no VME or ME and a 4.7% of MI was found which was derived from 4 *K. pneumoniae* with close-to-breakpoint MICs that fell in the S (*n* = 2, MIC = 2 mg/l) or I (n = 2,) category by BMD-Difco but were categorized as I or R by MHB from Condalab respectively.

### Evaluation of performance of Etest in relation to BMD-Difco

Comparison of TGC MIC results obtained by Etest showed a high level of agreement with those obtained using BMD (CA 98.8%, EA 100%) (Weighted kappa = 0.976, SE = 0.024, 95% CI = 0.929–1.00). Overall, Etest MIC values were either identical (61.9% isolates, *n* = 52 isolates, 17 *E. coli*, 18 *K. pneumoniae*, 17 *A. baumannii*) or within − 1(29.7%, *n* = 25 isolates, 12 *E. coli*, 11 *K. pneumoniae*, 2 *A. baumannii*) or + 1 doubling dilution (8.3%, *n* = 7 isolates, 6 *A. baumannii*, 1 *E. coli*) of the BMD MICs. There were no categorical discrepancies between the two testing methodologies except for one *K. pneumoniae* isolate with close-to-breakpoint MIC (=8 mg/l) which was categorized as R by BMD but I by the Etest resulting in overall MI rate of 1.19% (Table 2).

**Table 2:** Performance characteristics of different TGC susceptibility testing methods compared to broth microdilution performed using Mueller Hinton Broth from Difco

Method and isolate group	No. (%) of isolates					
	Susceptible	Intermediate	Resistant	EA (%)	CA (%)	MI n(%)
BMD1						
All isolates	68 (80.9)	7 (8.3)	9 (10.7)			
*K. pneumoniae*	19 (65.5)	2 (6.8)	8 (27.5)			
*A. baumannii*	19 (76)	5 (20)	1 (4)			
*E. coli*	30 (100)	0	0			
BMD2						
All isolates	66 (78.5)	7 (8.3)	11 (13)	100	95.23	4 (4.76)
*K. pneumoniae*	17 (58.6)	2 (6.8)	10 (34.4)	100	86.20	4 (13.7)
*A. baumannii*	19 (76)	5 (20)	1 (4)	100	100	0
*E. coli*	30 (100)	0	0	100	100	0
Etest						
All isolates	68 (80.9)	8 (9.5)	8 (9.5)	100	98.80	1 (1.19)
*K. pneumoniae*	19 (65.5)	3 (10.3)	7 (24.1)	100	96.55	1 (3.4)
*A. baumannii*	19 (76)	5 (20)	1 (4)	100	100	0
*E. coli*	30 (100)	0	0	100	100	0
AD						
All isolates	58 (69)	11 (13)	15 (17.8)	77.38	80.95	16 (19)
*K. pneumoniae*	17 (58.6)	2 (6.8)	10 (34.4)	96.55	86.20	4 (13.7)
*A. baumannii*	11 (44)	9 (36)	5 (20)	36	52	12 (48)
*E. coli*	30 (100)	0	0	93.33	100	0
DD						
All isolates	68 (80.9)	3 (3.5)	13 (15.4)	NA	95.23	4 (4.76)
*K. pneumoniae*	19 (65.5)	2 (6.8)	8 (27.5)		100	0
*A. baumannii*	19 (76)	1 (4)	5 (20)		84	4 (16)
*E. coli*	30 (100)	0	0		100	0

*NA* Not applicable, *EA* Essential agreement, *CA* Categorical agreement, *MI* Minor error, *BMD1* Broth microdilution –Difco, *BMD2* Broth microdilution -Condalab, *AD* Agar dilution, *DD* Disk diffusion

### Evaluation of performance of agar dilution in relation to BMD-Difco

Overall, for 91.6% of strains (*n* = 77) MICs determined by agar dilution were at least 2-fold higher than those determined by reference BMD resulting in poor correlation between two methods (overall EA:77%, CA:81%) (Weighted kappa = 0.697, SE = 0.067, 95% CI = 0.566–0.829) and unacceptably higher MI rate (19%). Despite the poor intermethod agreement, no ME or VME was detected (Table 2). The agreement between two methods differed on a species dependent manner, exceeding the acceptable performance rate for AST methods (> 90%) for *K. pneumoniae* and *E. coli* but being found unacceptable for *A. baumannii* isolates. Indeed, the performance of AD was the best for determination of MIC in *E. coli* where MICs were identical (*n* = 4), + 1(*n* = 24 isolates) or + 2 (*n* = 2 isolates) doubling dilution of BMD MICs.

### Evaluation of performance of disk diffusion in relation to BMD-Difco

**Fig. 2:**
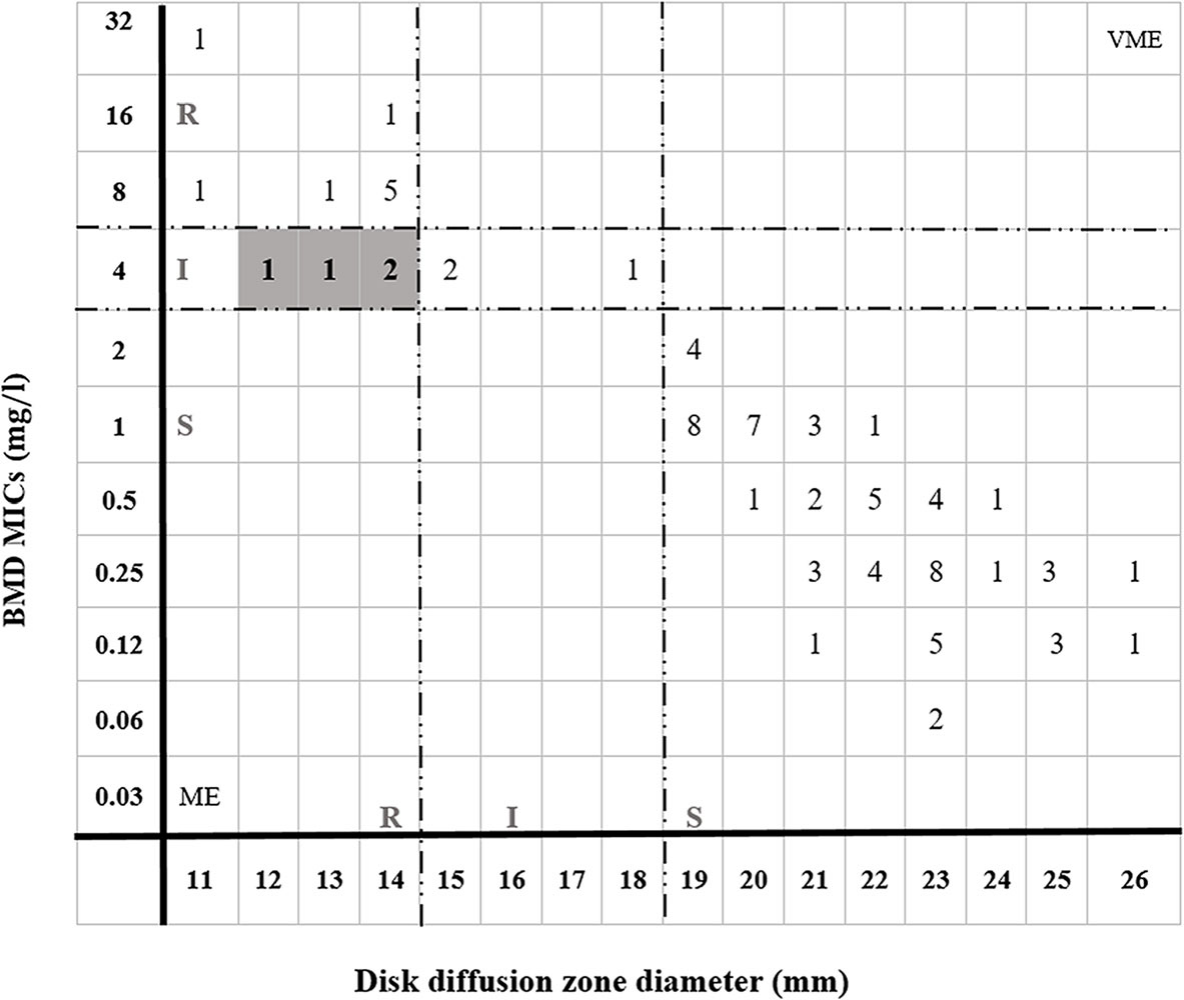
Scattergram of tigecycline zone diameters versus BMD MICs for 84 clinical isolates of gram-negative bacteria

Overall the inhibition zone diameter observed among the isolates ranged from 19 to 26 mm among TGC-S, 12–18 mm among TGC-I and 11–14 mm among TGC-R isolates. DD correctly detected all TGC-S (*n* = 68) and TGC-nonsusceptible (*n* = 16) isolates identified by reference method which resulted in high level overall CA between two methods (95%) (Weighted kappa = 0.911, SE = 0.040, 95% CI = 0.833–0.990). While the performance of DD for determination of TGC susceptibility among Enterobacteriaceae was excellent, (CA:100% with no errors), the CA was slightly lower when this method was used for *A. baumannii* (84%) where it was associated with an unacceptably high minor-error rate (16%) (Table 2). All the minor errors were intermediate findings interpreted as resistant (*n* = 4 intermediate isolates having inhibition zone diameter of less than 15 mm (12 mm (*n* = 1), 13 mm (*n* = 1), 14 mm (*n* = 2)) (Fig. 2). This could be attributed to the fact that the susceptibility of *A. baumannii* was categorized based on the FDA susceptibility breakpoints for Enterobacteriaceae.

## Discussion

The emergence of multi-drug resistance among nosocomial GNB greatly limits therapeutic options and necessitates the use of last resort antibiotics including TGC for the treatment of life-threatening infections. Therefore, there is an increased need for reliable methods to predict the clinical outcomes adequately. The TGC susceptibility testing is plagued by problems including the lack of predictive breakpoints for interpretation of the results in CLSI (although the EUCAST breakpoints are only available for few GNB (*E. coli* & *C. koseri*)) and the variety of factors that can affect the susceptibility testing results. The U.S. FDA susceptibility breakpoints are used by many laboratories for interpretation of the results. However, there are no existing susceptibility breakpoints for some of the most important clinical pathogens including *A. baumannii* from any breakpoint organization, even in FDA. It has been shown that aged broth may lead to misleadingly higher TGC MIC values for some isolates and it is postulated that this may be due to inactivation by dissolved oxygen [12]. Therefore, it is recommended that broth MIC determinations for tigecycline should always be performed using fresh media (< 12 h after autoclaving). Also a previous study found different MIC values obtained by Etest performed using Mueller-Hinton media from different manufacturers (Oxoid, Bio-Rad, BD) for all tested organisms [14]. Others reported obtaining higher TGC MICs performed by Etest using Mueller-Hinton agar from Merck compared to MHA from either Oxoid or Difco. The differences were attributed to a much higher concentration of manganese in the medium from Merck compared to Difco or Oxoid [13]. We conducted the present study to assess the performance of different susceptibility testing methods against the reference BMD using the FDA breakpoints. All methods were performed using MHA/B from one manufacturer (Difco), except for BMD which was performed using MHB from two different suppliers (Difco and Condalab) to check the effect of medium type on TGC MICs. While comparing MICs obtained by BMD performed using MHB from two suppliers, revealed a good correlation (acceptable EA and CA), we found that MHB from Condalab produced a 2-fold dilution higher MICs than BMD-Difco for 50% of the isolates. This rise in MIC did not result in change in categoric susceptibility of most of the isolates except for those with MICs close to susceptibility breakpoints (MIC = 2 or 4 mg/l) which led to a slightly higher overall MI rate (4.7%). The identified minor discordance between MICs obtained by using the two MHB media may be attributed to variation in manganese content of the medium from each manufacturer. Determination of manganese content of the medium using methods such as “inductively coupled plasma optical emission spectrometry” or “atomic absorption spectroscopy”, was not possible in the current   study and this information was not available from the Condalab company for the batch we used. However, it is  speculated that, the higher MIC values obtained by MHB from Condalab may possibly be linked to a higher manganese concentration in this medium compared to reference medium. Among the studied methods, Etest showed the best correlation with BMD, being characterized with the lowest error rates and highest EA and CA. For the majority of isolates (91.6%) Etest yielded either identical or one doubling dilution lower MICs than BMD MICs except for *A. baumannii* where Etest produced higher MICs among 24% of isolates. Other studies have reported obtaining higher or lower MICs given by Etest compared to MICs determined by BMD depending on bacterial isolate tested where higher Etest MICs were noted among *A. baumannii* isolates compared to BMD MICs [21]. Etest has also been reported in a previous study to yield ≥1-fold dilution lower MIC values compared to BMD MICs when evaluated for testing TGC susceptibility of Enterobacteriaceae [22].

AD yielded the least correlation with BMD among the studied methods. This discordance was reflected in a low essential agreement between the two methods in a bacterial isolate dependent manner. The most apparent difference between the two testing methodologies was noted to occur among the A*. baumannii*, where a two (*n* = 8 isolates) to four fold (*n* = 13 isolates) or higher increase (eight fold, *n* = 3 isolates) in AD MICs was found resulting in low CA and EA and higher MI relative to BMD. However, an acceptable EA (96, 93%) or slightly acceptable CA (86, 100%) was obtained when AD MICs were compared to BMD MICs among Enterobacteriaceae. Torrico et al., who tested the TGC susceptibility of Enterobacteriaceae using 3 different testing methodologies (BMD, Etest and AD), also reported obtaining the highest MIC values by AD compared to other methods [22]. While DD showed excellent agreement with BMD in terms of correct categorization of all TGC-S, TGC-I and TGC- R isolates of *E. coli* and *K. pneumoniae*, it produced unacceptably higher minor error rate (16%) for *A. baumannii* which probably stemmed from applying the FDA tigecycline breakpoints issued for Enterobacteriaceae for data interpretation in this bacterium. This is in agreement with a previous study which found higher MI for DD compared to BMD among ESBL-producing *K. pneumoniae* and *A. baumannii* isolates [23]. Moreover, testing the TGC susceptibility of *Acinetobacter* spp. by DD in another study also revealed a high minor-error rate (23.3%) (by referring to FDA Enterobacteriaceae breakpoints for data interpretation). The adjustment of breakpoints (susceptible/resistant) to ≥16/≤12 mm by the authors improved the intermethod agreement and minimized error rates down to 9.7% [24]. In fact, the validation of DD breakpoints for *A. baumannii* is needed to improve the performance and predictive value of this method. With a present global increase of TGC-resistance among *A. baumannii* isolates, performing a DD assay may be the most practical and available method in many laboratories, especially in those of developing countries.

In conclusion, with regard to BMD performed using MHB from Condalab and Etest we found MIC values which differed by one doubling dilution in 50 and 38% of isolates from reference method MICs (BMD-Difco) respectively. Since these discrepancies in MICs can affect the clinical classification of isolates with close-to-breakpoint MICs, testing the susceptibility using a reference AST method or medium with optimized concentration of manganese is recommended if obtained MICs are found to be close to susceptibility breakpoints. Despite the lack of detected VME or ME, DD and AD should not be used for routine TGC susceptibility testing of *A. baumannii,* due to poor correlation with BMD. The higher intermethod MI error rate obtained for DD in *A. baumannii* isolates increasing a need for adjustment of susceptibility breakpoints by the breakpoint organizations. In general, all testing methods showed acceptable (> 90%) EA and/or CA with BMD in *E. coli* isolates which could stem from the fact that TGCs MIC ranges in these bacteria were very low and none of isolates were characterized with close-to-breakpoint MICs.

## Data Availability

The datasets used and/or analyzed during the current study are available from the corresponding author on reasonable request.

## Abbreviations

TGC: Tigecycline
GNB: Gram-Negative bacteria
R: Resistant
I: Intermediate
S: Susceptible
TGC-S: Tigecycline susceptible
TGC-NS: Tigecycline non-susceptible
AD: Agar dilution
DD: Disk diffusion
BMD: Broth microdilution
EA: Essential agreement
CA: Categorical agreement
MI: Minor error
ME: Major error
VME: Very major error
MIC: Minimum inhibitory concentration
FDA: Food and drug administration
MHA: Mueller-Hinton Agar
MHB: Mueller Hinton broth
